# Supplementary figures and images for: Whole blood transcriptomics analysis of Indonesians reveals translocated and pathogenic microbiota in blood
Source: PLoS One. 2025 Jul 24;20(7):e0328788. doi: 10.1371/journal.pone.0328788 (PMC12289045; doi:10.1371/journal.pone.0328788)

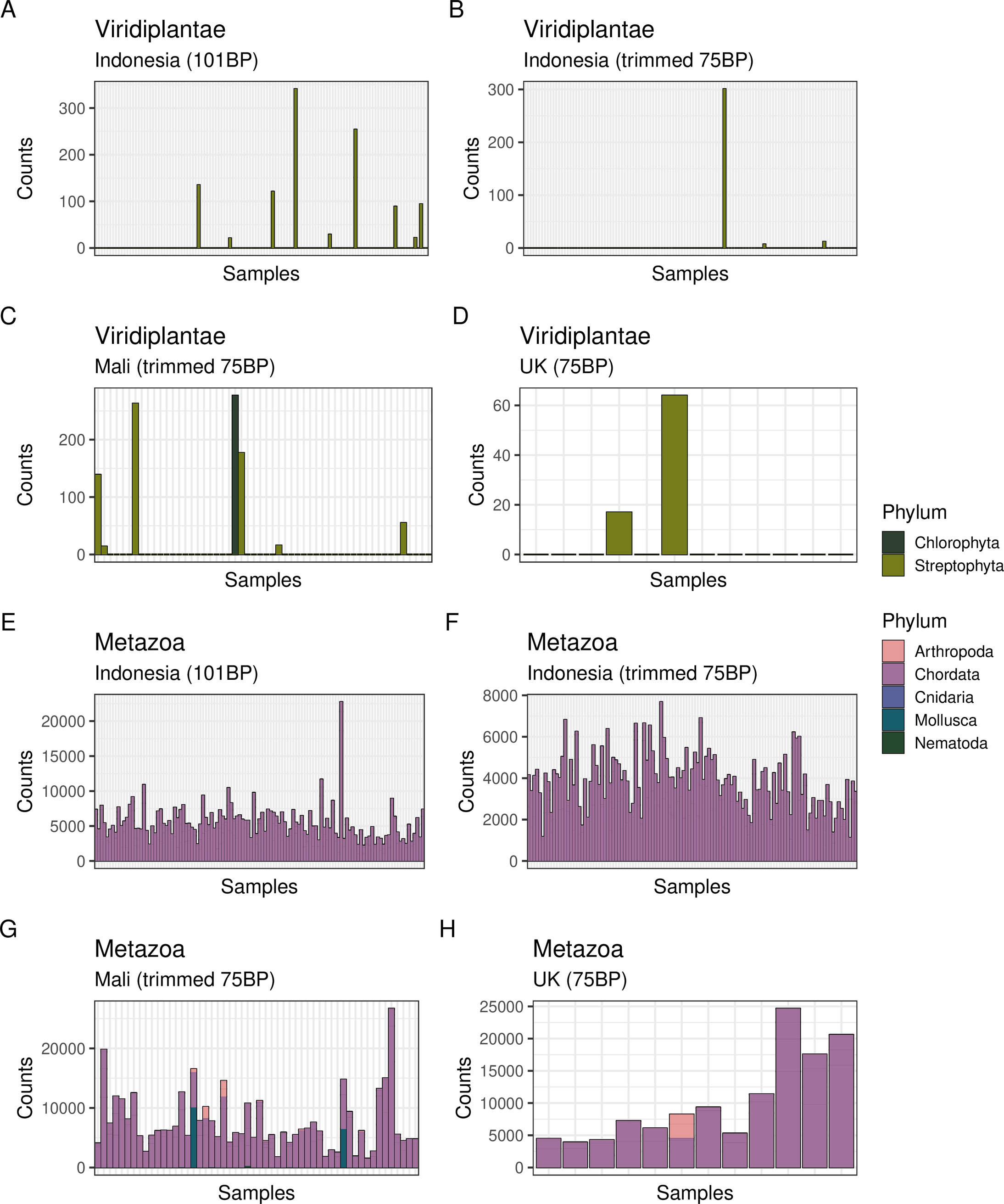

Supplement: S1 Fig — A–D) Reads mapping to the Viridiplantae E–H) and Metazoa. (TIF) [file pone.0328788.s001.tif]

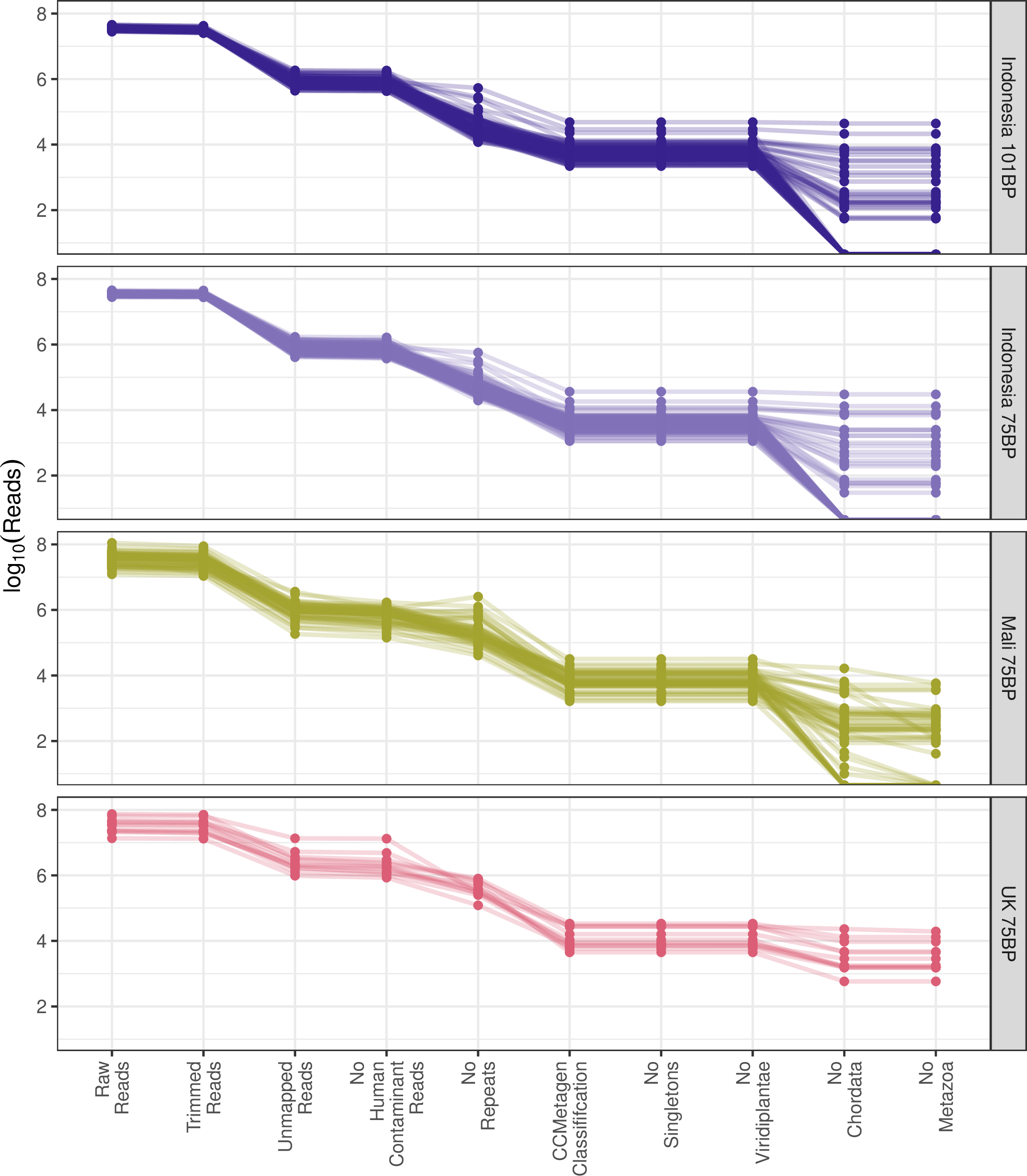

Supplement: S2 Fig — (TIF) [file pone.0328788.s002.tif]

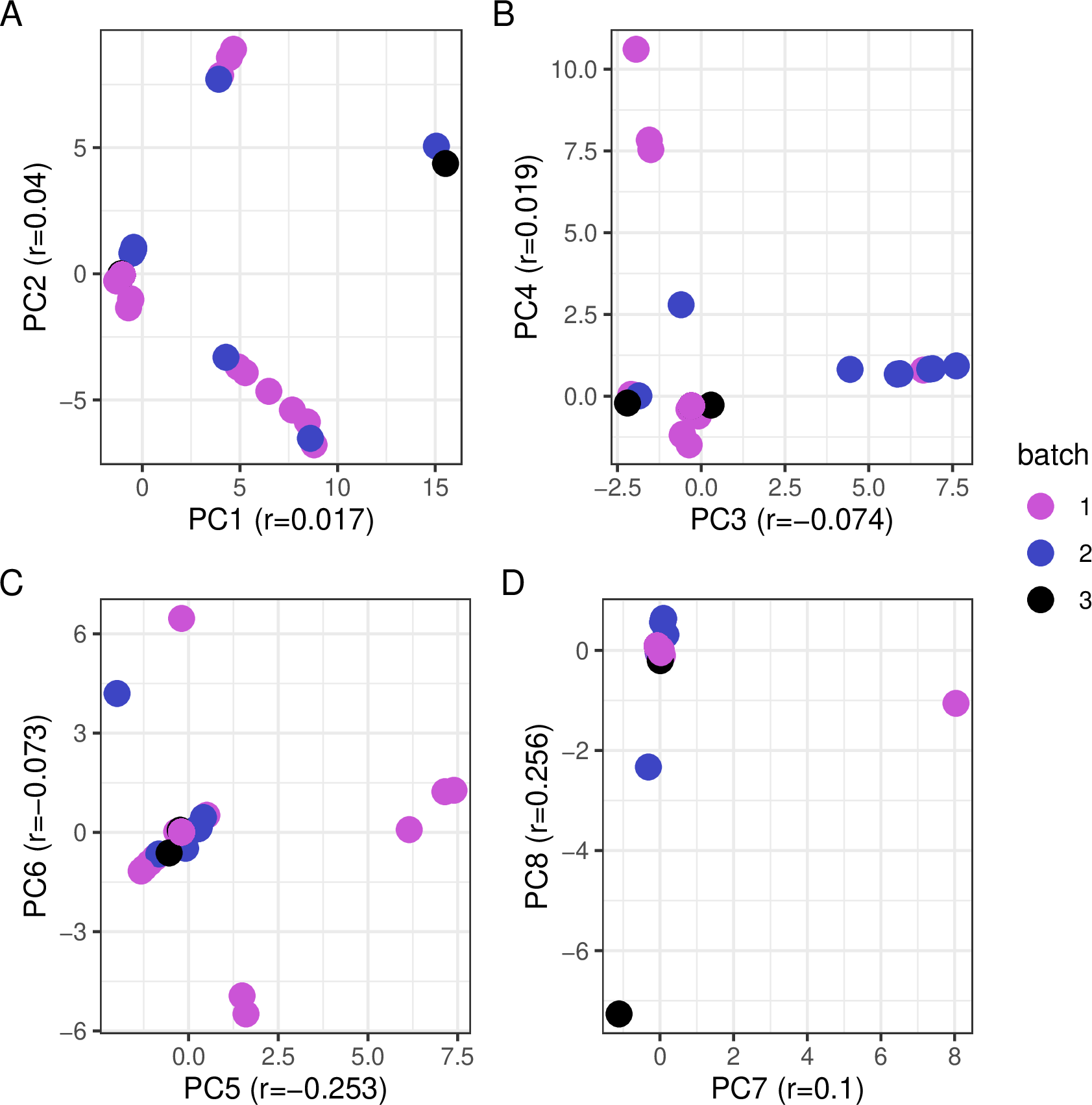

Supplement: S3 Fig — No clear clustering and strong Spearman correlation (r) were seen between batch and A) PCs 1-2, B) PCs 3-4, C) PCs 5-6, and D) PCs 7-8. (TIF) [file pone.0328788.s003.tif]

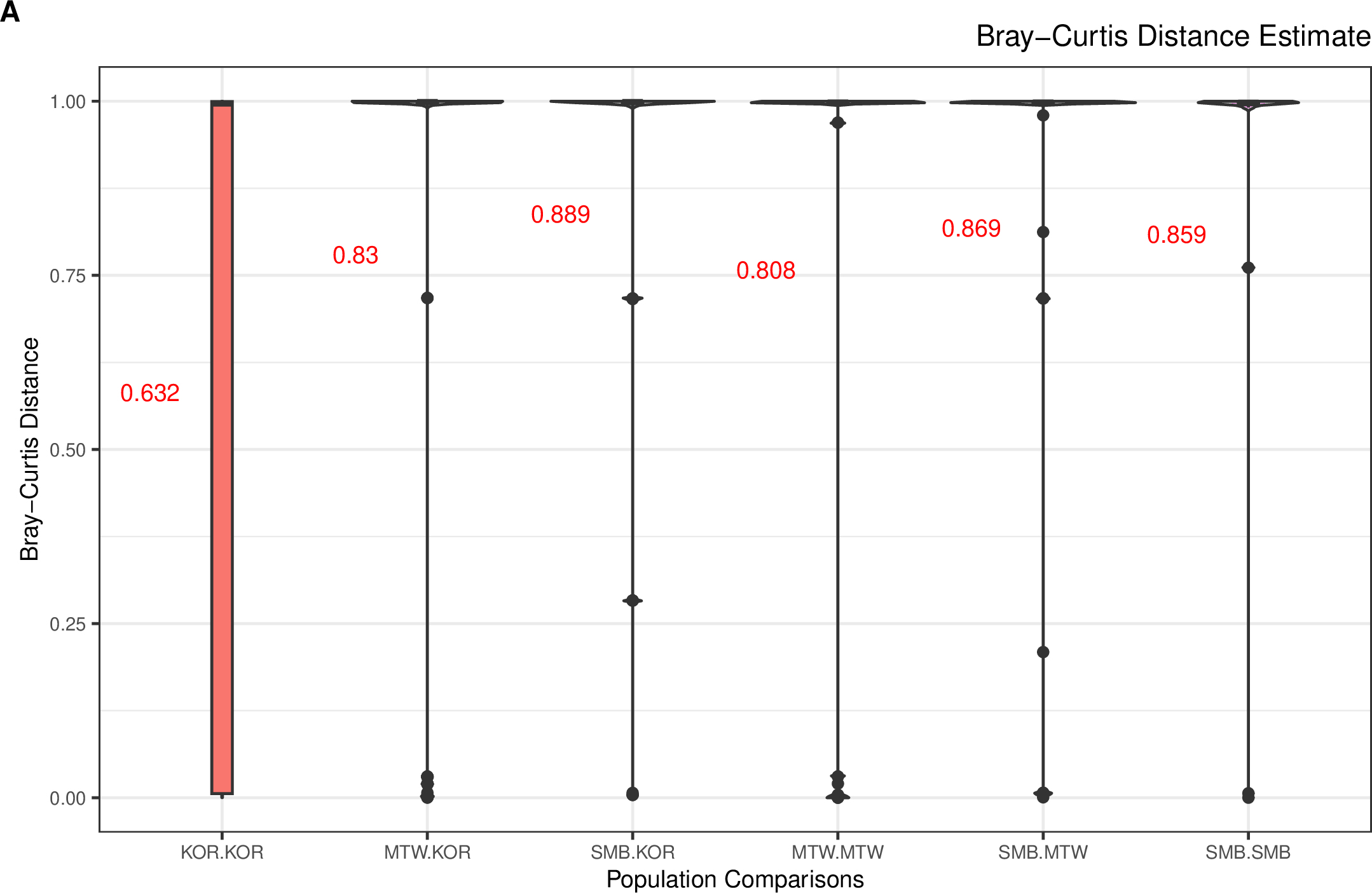

Supplement: S4 Fig — (TIF) [file pone.0328788.s004.tif]

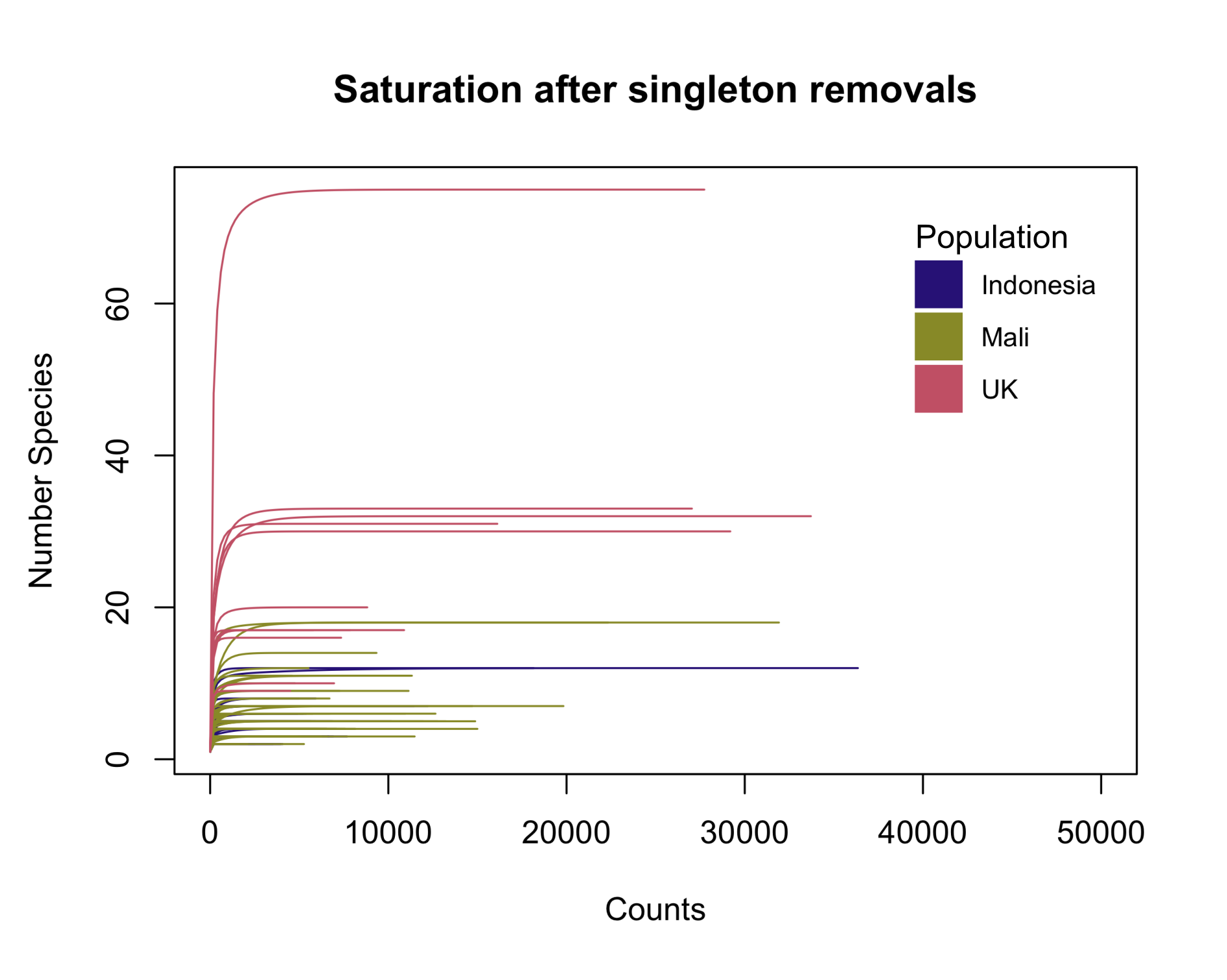

Supplement: S5 Fig — (TIF) [file pone.0328788.s005.tif]

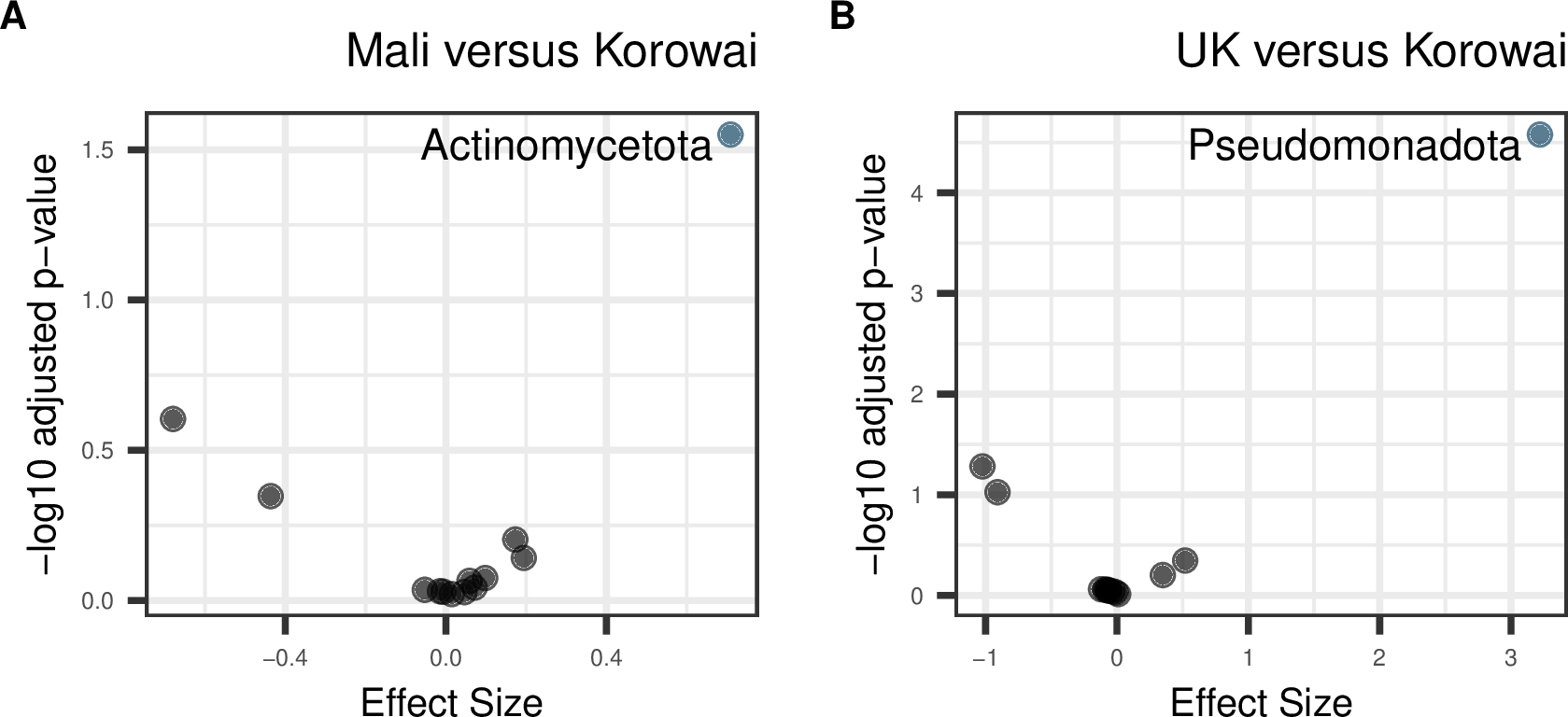

Supplement: S6 Fig — A) Volcano plot of BH adjusted p-values from Welch’s t-test for each phyla in the Korowai versus Malian populations and B) Korowai versus UK populations. Taxa with a BH-corrected p-value below 0.05 for are coloured by superkingdom (blue: bacteria). (TIF) [file pone.0328788.s006.tif]

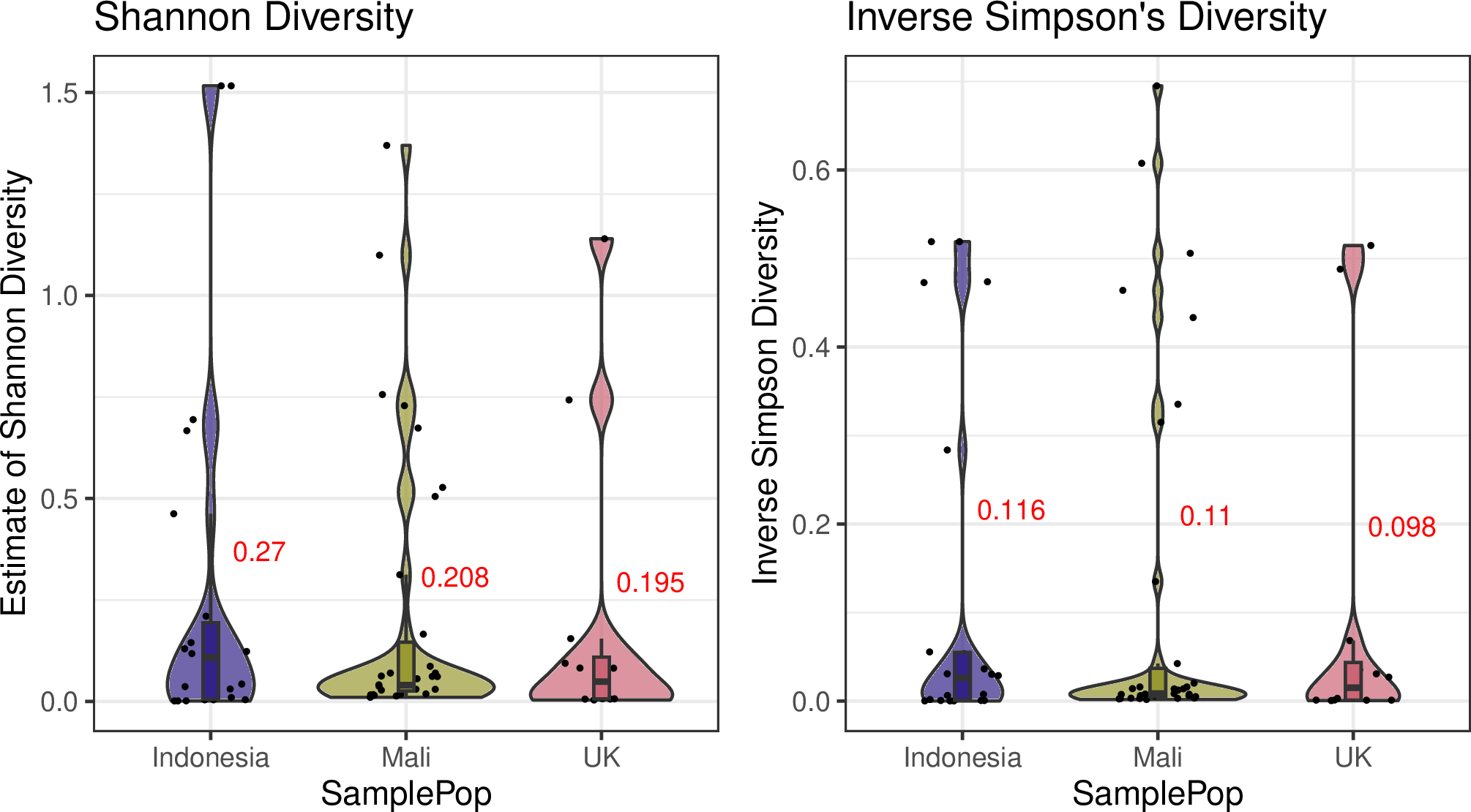

Supplement: S7 Fig — (TIF) [file pone.0328788.s007.tif]
