# Supplementary material for: Whole blood transcriptomics analysis of Indonesians reveals translocated and pathogenic microbiota in blood
Source: PLoS One. 2025 Jul 24;20(7):e0328788. doi: 10.1371/journal.pone.0328788 (PMC12289045; doi:10.1371/journal.pone.0328788)
Supplement: Attachment — Submitted filename: 126657_1_rebuttal_3051114_sf265p.docx [file pone.0328788.s016.docx]

**Manuscript number:** RC-2022-01449

**Corresponding author(s):** Irene, Gallego Romero

*[Please use this template only if the submitted manuscript should be considered by the affiliate journal as a full revision in response to the points raised by the reviewers.*

*If you wish to submit a preliminary revision with a revision plan, please use our "*[*Revision Plan*](https://docs.google.com/document/u/0/d/1HHp62Tfeb44nd3Gu_3ChB0b8gmMdEog7GjZcZFwHSiQ/edit)*" template.* ***It is important to use the appropriate template to clearly inform the editors of your intentions.****]*

## 1. General Statements [optional]

*This section is optional. Insert here any general statements you wish to make about the goal of the study or about the reviews.*

We have carefully reanalyzed the data in response to more recent findings regarding what constitutes a core blood microbiome in healthy humans. This results in fewer taxa to report compared to our previous findings, which rendered the second half of our manuscript (the ICA analyses) less significant. We therefore decided to remove that part. We have updated all the figures and supplementary files accordingly and are confident in the robustness of our findings from the rest of our analyses.

## 2. Point-by-point description of the revisions

*This section is mandatory.* *Please insert a point-by-point reply describing the revisions that were already carried out and included in the transferred manuscript.*

**(Our response is colored in dark green).**

Reviewer #1 (Evidence, reproducibility and clarity (Required)):

Summary: Authors performed a metatranscriptomic analysis from publicly-available datasets of whole blood from 3 places in Indonesia. Their goal was to explore which pathogens were present on the blood of those 117 healthy individuals. It was interesting that reads from Flaviviridae and Plasmodium were detected in asymptomatic subjects.

Major comments:
1) How did the authors assess and correct batch-effects between different datasets?

**Our response:** We have sequencing batch information for the Indonesian dataset and saw no clear clustering based on batches in the first 8 PCs. We recognize that sampling variations may exist between islands, though the taxa matrix we acquired from the unmapped reads are very scarce that such variations did not have a strong enough effect to introduce batch effects in our microbiome analyses, and that the signals were driven by pathogenic reads. For our comparative analyses between datasets, we made sure that all three datasets shared similar processing (collected using Tempus Blood RNA Tubes and went through globin depletion method) and have trimmed both Indonesian and Malian reads to match the length of the UK reads (75BP).

2) Did the RNA-seq capture poly-A mRNAs? If so... these reads that did not map the human genome were captured because of internal priming. Can they find internal poly A sequences in the genome of Flaviviridae and Plasmodium pathogens? I would like to know that to understand the source of the reads and which other pathogens may be missing (due to the lack of internal priming).

**Our response:** No, our dataset did not capture poly-A mRNAs. We performed ribosomal RNA (rRNA) and globin mRNA depletion.

3) Principal coordinates analysis (PCoA) is often utilized in metagenomics analysis. Although they are equivalent, is there a reason for using PCA?

**Our response:** Since we used CLR transformation, the resulting matrix lies in Euclidian space. PCA is just a form of PCoA in Euclidian space.

Minor comments:
1) "Indonesia is a country with large numbers of endemic and emerging infectious diseases [16], making it a crucially important location to monitor and understand the effects of pathogens on human hosts." Is there any epidemiological data that shows differences in infectious diseases across these 3 places? Can the authors provide a map and better explanation about the importance in comparing these 3 areas?

**Our response:** We have added references to malaria infection being more prevalent in the eastern side of Indonesia in the discussion section.

2) Why is it so hard to try to identify (only for Flaviviridae reads) reads that map to very relevant viruses, such as Zika, Dengue, and Yellow Fever? Why did the authors state that they "were unable to refine this assignment further" if this is one of the most interesting finding?

**Our response:** Our reanalysis showed a small percentage of the Flaviviridae reads to be assigned to the *Pegivirus* genus. As more diverse microbial genomes are added to reference databases and identical regions become more common between them, it becomes harder for the classifer to further define reads to species level (<https://link.springer.com/article/10.1186/s13059-018-1554-6>). Flaviviridae has distinct species spread across six different genera (<https://www.ncbi.nlm.nih.gov/Taxonomy/Browser/wwwtax.cgi?id=11050>). In comparison, despite Plasmodiidae having more species recorded compared to Flaviviridae, an overwhelming majority of the species is part of the *Plasmodium* genus, hence we were able to refine them down to species-level (<https://www.ncbi.nlm.nih.gov/Taxonomy/Browser/wwwtax.cgi?id=1639119>).

3) Is the script available at https://gitlab.unimelb.edu.au/igr-lab/Epi_Study ? This reviewer could not access it.
**Our response:** We thank Reviewer 1 for pointing this out and have amended the link, now accessible here: <https://gitlab.svi.edu.au/muhamad.fachrul/indo_blood_microbiome>

Reviewer #1 (Significance (Required)):

Interesting paper that enable to extract additional knowledge from whole blood RNA-seq data. There are already several papers that do this and I think authors could go one step forward (for instance, PCR validation of additional individuals). I don't think this can be used for surveillance if it cannot identify species, it is more expensive than running targeted assays, and that may be many false negative pathogens in the samples.

**Our response:** We thank Reviewer 1 for their comments. We have updated our manuscript to reflect our updated analyses which minimizes false positive taxa and the project’s significance not as a mainline surveillance tool, but a retrospective one.

Reviewer #2 (Evidence, reproducibility and clarity (Required)):

Summary:

Bobowik and colleagues perform a computational analysis of whole blood RNA-seq datasets from healthy individuals of three different regions of Indonesia. Their goal is to identify infecting pathogens and other microbes and correlate their abundances to host gene expression patterns or health characteristics in these populations. They find a broad range of bacterial, viral and microeukaryote taxa. When comparing the three Indonesian populations, they find that the Korowai population is the most diverse and different from the other two, possibly driven by the higher prevalence and abundance of Plasmodium (Apicomplexa) in this population.

Then, the authors conduct a statistical decomposition of human gene expression in these samples in independent factors using ICA, and correlate each of these factors to the abundances of the microbial taxa detected. This analysis allows researchers to associate specific patterns of gene expression, such as immune-related pathways, to the presence of members of the Apicomplexa and Kitrinoviricota phyla.

Lastly, the authors use previously published data from other two cohorts (from Mali and the UK) to contextualize their blood microbiome findings. They find microbial reads in all datasets. The Mali cohort is characterized by a large abundance of archaea, not found in the other two populations, while the UK cohort has the lower diversity. Altogether, the authors propose the use of RNA-seq data from human whole blood as a way to study the blood microbiome and establish potential associations between blood resident microbes and host gene expression

Major comments:

1. The methodology to filter and remove reads from potential contaminants needs to be more stringent to ensure the results do not contain spurious contaminants and that the conclusions are correct. It has been described that genomic databases are heavily contaminated with human sequences (Steinegger and Salzberg, 2020), and in this manuscript, even after a two-pass alignment with STAR, reads mapping to helminths also corresponded to the human genome. Additionally, ad-hoc removal of specific taxa (Metazoa and Viridiplantae) was only performed after suspicion of contamination. However, this ad-hoc removal cannot be performed with microbial (bacterial, viral, etc.) contaminants as there is a risk of removing actual bacteria from the samples. But it has been confirmed that many microbial assemblies also suffer from human contamination. Possible actions to take are the following:
a.Perform the human mapping with more lenient parameters to avoid human reads to map to other (likely contaminated) genomes in genome databases.
b.Remove common contaminants that have been documented, for instance in blood (Chrisman et al., 2022).
c.Run a tool to detect contaminated contigs in the database used to map reads to microbes and remove these problematic contigs from further analysis.

**Our response:** We thank Reviewer 2 for the suggestions, especially to address contaminants. We have reanalyzed our data which resulted in much fewer taxa yet still retained the main pathogenic findings.

2. In line with the above, removing singletons (as I have understood these are taxa that are represented by a single read), is a way to minimize the risk of contamination. To take advantage of the functional profiling of RNA-seq, a measure to ensure that microbes found in blood are active would be to include in the analysis only taxa for which expression of more than a few genes is detected. This type of filtering has been previously applied in studies where very low microbial loads are expected (Lloréns-Rico et al., 2021). In this study, it has only been applied to the specific case of the archaeal taxon Methanocaldococcaceae. However, I would expect cleaner results if applied consistently to all taxa detected.

**Our response:** We have reanalyzed the data and applied this to all taxa detected.

3. The specificity of Methanocaldococcaceae in the samples from Mali is very striking. I am highly suspicious that this only occurs due to a batch effect, even though the authors were highly selective in their cohorts to avoid these. In fact, I extracted the genes spanning the regions highlighted in Supplementary Figure 9 of the Methanocaldococcus jannaschii genome. A BLAST search of these sequences returned, among Methanocaldococcus hits, hits from the ERCC synthetic spike-in sequences, used as internal controls in many RNA-seq experiments. ERCC synthetic spike-in hits appeared for all 4 regions in the genome of M. jannaschii highlighted in this figure. In the original publications of this dataset, there is no reference to the use of these ERCC controls, but given the observed matches, I suggest the authors to perform an extra step in their filtering pipeline to remove all reads mapping to these ERCC standards in all their three cohorts to prevent these sort of batch effects.

**Our response:** We thank Reviewer 2 for pointing this out. Our reanalysis, which now used proper 2-pass mapping and further downstream classification with both pairs of the reads, no longer detected any archaea.

4. I am puzzled by the inconsistencies shown between forward and reverse reads when mapping paired-end data. I expect these inconsistencies at lower taxonomic ranks (species or genus level) due to incomplete genomes, but not at higher taxonomic ranks. I wonder if, by performing more stringent filtering of contaminants as suggested above, the consistency between forward and reverse reads increases and both mates can be used, making the mapping more reliable.

**Our response:** We have reanalyzed the data using both pairs of the reads for classification, resulting in less detected taxa. We believe the new results are more robust as it no longer includes taxa that are not typically found in humans (such as the archae Methanocaldococcus and other environmental bacteria).

In summary, my main concerns regarding this manuscript involve the possibility that contaminants in the sequencing data may be the cause of some of the results presented, and I tried to propose ways of dealing with these contaminants. While some of the results may not be affected by detection of contaminants (i.e. the association between Apicomplexa and some ICs), others such as the diversity measures or the comparison across cohorts may be severely affected. I will consider these results highly preliminary until a more thorough and stringent approach for contaminant removal is applied.

**Our response:** We thank Reviewer 2 for the suggestions and have updated our manuscript with results updated analyses that are more stringent towards contaminants, as can be seen from our updated findings.

Minor comments:

1. I would appreciate some of the analyses done at lower taxonomic levels if the sparsity of the data allows it, after removing contaminants. Given that the CLR transformation does not allow for zeros, other alternatives such as GMPR (Chen et al., 2018) or adding a pseudocount would allow these analyses?

**Our response:** After our reanalysis, we ended up with even sparser data and therefore could not perform the analyses at lower taxonomic levels.

2. In the PCA shown in figure 1, does the number of microbial reads detected correlate with any of the first two components?

**Our response:** Yes Plamosdiidae correlates well with PCs 1 and 2 (0.66 & 0.73) and Flaviviridae correlates very strongly with PC1 (0.917). We have added this detail in the results section.

3. In Figure 1C, the x axis is wrongly named PC2.

**Our response:** We thank Reviewer 2 for pointing this out and have amended this detail.

4. There is a typo in the legend of Figure 1A ("showeing")

**Our response:** We thank Reviewer 2 for pointing this out and have amended this detail.

5. In the alpha diversity estimates comparison across the three different cohorts, after subsampling each population to achieve similar sample size in each cohort, it is stated that "after subsampling, each population had similar diversity estimates". However, the numbers shown afterwards corresponding to the mean values of alpha diversity, without confidence intervals or a boxplot/violin plot together with an accompanying statistical test, are not enough to assess similarity. I would appreciate a figure (similar to Figure 3E and F) or a test accompanying these mean values.

**Our response:** We thank Reviewer 2 for pointing this out and have amended this detail.

6. In the volcano plots (Figure 3A, B and others throughout the manuscript) it would help the reader to add lines for the thresholds chosen for the effect size and -log10(p-value) to separate significant results.

**Our response:** We thank Reviewer 2 for pointing this out and have amended this detail.

7. In Figure 3E and F, I would appreciate having bars for the statistically significant comparisons.

**Our response:** We thank Reviewer 2 for pointing this out and have amended this detail.
