# Supplementary material for: Whole blood transcriptomics analysis of Indonesians reveals translocated and pathogenic microbiota in blood
Source: PLoS One. 2025 Jul 24;20(7):e0328788. doi: 10.1371/journal.pone.0328788 (PMC12289045; doi:10.1371/journal.pone.0328788)
Supplement: Attachment — Submitted filename: response_to_editor_reviewers_PLOSONE_EiC_02052025.docx [file pone.0328788.s017.docx]

## 1. Point-by-point description of the revisions

Editor:

a) Please explain how the author relates this study's impact to John P. Hanley et al. (2021) and Louis et al. (2005). What is the significance of whole blood transcriptome analysis, which identified distinct gene sets that correlated with viremia and revealed varying gene expression patterns before and after infection, implying immune state changes?

**Our response:** We are not sure which exact papers the editor referred to, but the comment is not within the immediate scope of our manuscript as our study contains only pre-infectious individuals.

b) What are the hematological changes and cellular composition changes and Gene Expression Patterns in relational to host and pathogen?

**Our response:** Our previous works with this dataset have covered this, and we have cited them (references 25-27; <https://journals.plos.org/plosgenetics/article?id=10.1371/journal.pgen.1008749>, <https://www.sciencedirect.com/science/article/pii/S0002929721004377>, <https://www.biorxiv.org/content/10.1101/2021.01.07.425684v1>). In short, we found no significant difference in gene expression levels or cellular composition associated with pathogen loads in our cohort. This is not surprising as our cohort is comprised of self-reported healthy individuals, so even those with detectable malaria loads were not presenting symptoms.

c) Why did the author choose an RNA-based investigation while prior research using mNGS and shotgun metagenomics demonstrated a more extensive analysis?

**Our response:** We have mentioned in our abstract and discussion sections how this approach is not a one-to-one replacement for shotgun metagenomics as a main surveillance tool, but rather a valuable retrospective tool to look at transcriptomic data generated in the past with no matched metagenomic data available.

d) Author mentioned “Recent studies have found no evidence of a core microbiome circulating in the blood of healthy Stanley plot individuals” and “However, not enough studies have been done to confirm whether this lack of a core microbiome is also consistent in regions where infectious diseases are endemic” but plethora of literature is available depicting role of core microbiome circulating in the blood of healthy individuals –
e) Study by Païssé et al. (2016), Study by Castillo et al. (2019), Blauwkamp et al….. so on, Nature study by Ling et al. (2023) - large-scale study analyzed blood samples from 9,770 healthy individuals.

**Our response:** The consensus in the field is clearly converging on there being no core blood microbiome. We believe the Ling et al study mentioned by the editor is actually a reference to Tan et al. (2023) (<https://www.nature.com/articles/s41564-023-01350-w>), which emphasize the notion that the blood of healthy individuals do not support a consistent core microbial community. We cited this paper in our manuscript as reference number 19 (page 14, first paragraph of discussion in our manuscript) as it supports our argument. We also cited Castillo et al. (2019) as reference number 48, in which the paper presents the evidence of eukaryotes and viruses in blood transcriptome yet also challenges their presence in healthy human blood, not concluding their definite existence and encouraging researchers to be more careful with experimental design (which also supports our main argument).

This topic in the microbiome / metagenomics field has been, and remains highly debated, with new evidence challenging the accepted paradigm; the Païssé et al. (2016) reference the editor mentioned is already 8 years old and more recent and robust evidence (like the Tan et al. paper) have come out.

We’re unsure which Blauwkamp et al. paper the editor referred to as he did not specify the year, but the one in 2019 (<https://www.nature.com/articles/s41564-018-0349-6>) is not relevant to the author’s point in depicting circulating microbiome in healthy individuals.

f) Why does the author focus exclusively on Indonesia—specifically Mentawai, Sumba, and the Indonesian portion of New Guinea Island—as the data set for this study?

**Our response:** We have mentioned in the abstract and introduction how Indonesia is such an underrepresented population with high numbers of endemic and emerging infectious diseases, and why we specifically chose those three regions (*These populations span a gradient from west to east across Indonesia, thus capturing pathogens along the main geographical axis of the country).* There seems to be a fundamental misunderstanding of our manuscript by the editor, and a re-read or a different editor may help clarify this point.

g) Enhance the methodology section by providing a detailed description that emphasizes sample collection, RNA isolation procedures, library preparation, quantification, and host DNA depletion techniques. Include the catalogue information for the kits used to facilitate replication by others, as well as the parameters employed for the Illumina HiSeq 2500, and ensure appropriate citations are included.

**Our response:** This was already presented and provided in the original paper (which we’ve cited). It is not a new dataset and therefore would be weird to provide them again here.

h) Provide the consent information for all human individuals (Weight, Hight, BMI etc).

**Our response:** Consent is not related to anthropometric descriptors. Regardless, we’ve always cited the original paper the data was first presented in.

i) Provide the mapped and unmapped paired-end data separately?

**Our response:** This was already presented and provided in the original paper (which we’ve cited). It is not a new dataset and therefore would be weird to provide them again here.

j) Please verify that the given data files encompass all individual samples in the repository.

**Our response:** This was already presented and provided in the original paper (which we’ve cited). It is not a new dataset and therefore would be unfit to provide them again here.

k) Instead of a circular bar plot, the author should present a stanley plot that shows the interconnected changes between the samples; this will provide a better understanding of the data.

**Our response:** The editor is very unclear in their demands. There is no such widely acceptable type of plotting style as a “Stanley plot”. We simply do not understand what the editor meant by this. If the editor invented this type of plots, we are not aware.

l) For compositional data analysis, the author must execute isometric log-ratio (ILR) transformation, which preserves all metric aspects of the data, coordinates with a non-singular covariance matrix, and allows for simpler interpretation of results. Alternatively, utilise the Robust Centred Log-Ratio (rCLR) and Inter-Quartile Log-Ratio (IQLR) Transformations.

**Our response:** We have used the Centred Log-Ratio (CLR) transformation for our data, which is adequate for compositional data, as we have explained in the methods section (page 7, Sample clustering subsection).

m) The justification for doing Welch's t-test in this investigation is unclear author should use Limma-Voom, NOIseq, SAMseq, etc. for analysis?

**Our response:** The methods the editor suggested are not recommended for microbiome data. As explained in our method section, we used ALDEx2 to test for differences in species composition between populations, which applies CLR-transformation to correct for uneven library depth and data compositionality and uses Welch’s t-test for significance test (page 7, Differential abundance testing and diversity estimation subsection). We ran Welch’s t-test and default 128 Monte Carlo simulations for the phylum-level analyses, as ALDEx2 is more suited for lower-level classification (species, genus) differential abundance.

n) In discussion part author mentioned “Our understanding of pathogens found within remote regions of Indonesia, along with their impact on gene expression, is limited. But several published studies on global scenario showed impact of pathogens are associated with environmental factors like temperature, humidity, location of longitude Natri et al., 2020 etc. As author used European data in this study but some studies mentioned the commonalities in gene regulation between Indonesia and Europe (Natri wt al., 2022), So, instead of using European data, I strongly recommend that the author conduct a comparative and conclusive study using data from other continents.

**Our response:** We also included an African cohort (Mali) for validation, which resembles the endemic / rural environment of our Korowai donors more. Again, there seems to be a fundamental misunderstanding of our manuscript by the editor, and a re-read or a different editor may help clarify this point.

Reviewer 1:

The manuscript titled “The Whole Blood Microbiome of Indonesians Reveals Translocated and Pathogenic Microbiota” presents the use of transcriptomics data to explore the presence of pathogens in whole blood samples. The authors have responded to previous reviewer questions; however, a few minor comments are provided below:
1. If the authors agree, the title can be revised to "Whole Blood Transcriptomics Analysis of Indonesians…" to provide a clearer picture of the study.

**Our response:** We thank Reviewer 1 for the suggestion and have amended it.

2. If the primary aim of the study is to detect pathogens, a microbial-targeted metagenomic study could be considered. This approach may help address the issue of contaminated human reads that the authors encountered. Are there any previous studies that used metagenomic analysis for pathogen detection, and did they produce similar or different results? If using RNA-seq is more critical than metagenomics in this context, the authors could clarify this in the introduction or discussion section.

**Our response:** As mentioned in our abstract and discussion sections, our approach is more suited as a retrospective surveillance tool in cases where only past transcriptomic data are available with no matched metagenomic data or fresh samples, not as a main disease surveillance tool. We have cited a few studies using microbial-targeted metagenomic approaches for pathogens and mentioned them in the introduction, such as the study by Kafetzopoulou et al. (2019) who used Nanopore sequencing for Lassa virus (<https://pmc.ncbi.nlm.nih.gov/articles/PMC6855379/>).

Reviewer 2:

The manuscript under review focuses on a relevant but controversial topic: Assessing the state of the blood microbiome. I would like to focus on the ambiguity of the task that the authors set in their study. On the one hand, the presence of genome fragments of certain types of microorganisms in the blood was confirmed by experimental studies. On the other hand, the functional role of some of them remains unclear, especially in the context of the blood microbiome of healthy individuals. Although there is a point of view that there are no absolutely healthy people, the genomes of individuals are noted to be carriers of various potential pathological structures. Note that the authors used adequate methods for both obtaining data sets and bioinformatic and statistical processing to prove their hypothesis. In general, this manuscript is recommended for publication. I offer several technical recommendations:

**Our response:** We thank Reviewer 2 for their fair assessment.

1. The authors did not provide additional tables; therefore, their quality and necessity were not assessed.

**Our response:** We have provided 8 supplementary tables containing the read depth quality and differential abundance results of all the involved datasets. We understand if Reviewer 2 might not have received the supplementary files during the review process.

2. Methods, Datasets section, 1 paragraph: It is necessary to provide a reference to the citation in the text: "In the original Natri et al. study…».

**Our response:** We thank Reviewer 2 for pointing this out and have amended it.

3. Methods, Datasets section, paragraph 2: It is necessary to agree with the list of references and correct the author’s last name “The first dataset comes from Tran et al. [28, 29] …”.

**Our response:** We thank Reviewer 2 for pointing this out and have confirmed the author’s name is correct (Tuan M. Tran, hence Tran et al.).
