# Supplementary material for: Whole blood transcriptomics analysis of Indonesians reveals translocated and pathogenic microbiota in blood
Source: PLoS One. 2025 Jul 24;20(7):e0328788. doi: 10.1371/journal.pone.0328788 (PMC12289045; doi:10.1371/journal.pone.0328788)
Supplement: Attachment — Submitted filename: Comments_Bobowik_etal_PONE-D-24-24494-R1-June 2025.doc [file pone.0328788.s018.doc]

June 25, 2025

Manuscript PONE-D-24-24494R1

Comments on manuscript:

‘Whole blood transcriptomics analysis of Indonesians reveals translocated and

pathogenic microbiota in blood’

Bobowik et al.

This manuscript was previously commented on by another editor and two reviewers. The two reviewers had minor comments which were well answered in this first Rebuttal version. The comments from the editor met with difficulties for the authors to answer since there seem to be misunderstandings on what this manuscript aims to describe. As the new scientific editor I think that this is a well written and mostly comprehensive manuscript that describes interesting data on transcriptome (RNA seq) data on previously collected whole blood samples from 3 Indonesian populations. These data were compared to similar data from a population in Mali and a small population from London, UK.

I have one major and some minor comments to further improve the manuscript.

**Major comment**

As was also mentioned earlier by reviewer 2, Supplementary Tables 1 to 8 cannot be found in the document submitted to PlosOne (PONE-D-24-24494R1). Please provide these Suplementary Tables with the next Rebuttal version.

**Minor comments**

Abstract

It is mentioned that ‘two pathogens—Flaviviridae and Plasmodium—are the most predominantly abundant’. I suggest to change ‘pathogens’ into ‘taxa’ since that is more accurate. If the authors like they can add that these taxa includes pathogens such as Dengue viruses and malaria causing parasites.

In addition there are 5 ‘key highlights’ mentioned in the letter to the editor from May 2025. The first three key points deserve to be also mentioned in the abstract because in my view this is what the reader wants to know. There is no limitation in the number of words for an abstract for PlosOne, so this can be formulated it as comprehensive as needed.

Results

Supplemental Figure 1 and Figure 2: The legend colours and text do not everywhere line up; please adjust so these are aligned properly. For example ‘Chordata’ in Suppl Fig 1 is now next to a blue block whereas it should be next to a purple one. And ‘KOR’ should be next to the red blocks.

Supplementary Figure 5 contains interesting data which I think deserve to be shown in the main text. Please consider moving it there.
